# Resting-State vs. Task-Based Functional Magnetic Resonance Imaging in Neurosurgical Planning: A Narrative Review of Clinical Applications

**DOI:** 10.3390/biomedicines14071449

**Published:** 2026-06-26

**Authors:** Maurycy Rakowski, Natalia Anna Koc, Anna Dębska, Bartosz Szmyd, Agata Zawadzka, Karol Zaczkowski, Małgorzata Podstawka, Dagmara Wilmańska, Adam Dobek, Ludomir Stefańczyk, Dariusz J. Jaskólski, Karol Wiśniewski

**Affiliations:** 1 Faculty of Medicine, Medical University of Gdańsk, 80-210 Gdańsk, Poland; nataliaannakoc@gumed.edu.pl; 2 Department of Neurosurgery and Neurooncology, Barlicki University Hospital, Medical University of Łódź, 90-153 Łódź, Poland; anna.debska2@stud.umed.lodz.pl (A.D.); bartosz.szmyd@umed.lodz.pl (B.S.); karol.zaczkowski@umed.lodz.pl (K.Z.); malgorzata.podstawka@umed.lodz.pl (M.P.); dariusz.jaskolski@umed.lodz.pl (D.J.J.); karol.wisniewski@umed.lodz.pl (K.W.); 3 Siemens Healthcare sp. z o.o., 03-821 Warsaw, Poland; medycyna.pl@siemens-healthineers.com; 4 Department of Radiology and Diagnostic Imaging, Barlicki University Hospital, Medical University of Łódź, 90-153 Łódź, Poland; dagmara.wilmanska@umed.lodz.pl (D.W.); adam.dobek@umed.lodz.pl (A.D.); ludomir.stefanczyk@umed.lodz.pl (L.S.)

**Keywords:** resting-state fMRI, task-based fMRI, neurosurgical planning, presurgical functional mapping, brain tumors, language and motor mapping

## Abstract

**Background:** Accurate presurgical localization of eloquent cortex and subcortical pathways is essential in neurosurgery, guiding the balance between maximal safe resection and preservation of neurological function. This narrative review compares the clinical utility of task-based functional magnetic resonance imaging (tb-fMRI) and resting-state functional magnetic resonance imaging (rs-fMRI) in neurosurgical populations, with emphasis on brain tumors and epilepsy. **Methods:** This narrative review was based on a non-systematic literature search of PubMed/MEDLINE, Scopus, Web of Science, and Google Scholar from database inception to March 2026. The review focused on tb-fMRI and rs-fMRI for presurgical functional mapping in neurosurgical populations, including clinical utility, feasibility, validation, limitations, and workflow integration. **Results:** Tb-fMRI remains the most established noninvasive modality for motor and language mapping and language lateralization because of its task-specific activation maps and established role in clinical workflows. However, its use is limited by dependence on patient cooperation, task performance, and intact neurovascular coupling; thus, aphasia, cognitive impairment, fatigue, paresis, pediatric age, sedation, and tumor-related neurovascular uncoupling may render tb-fMRI inconclusive or misleading. Rs-fMRI offers a task-free alternative based on intrinsic functional connectivity, enabling simultaneous mapping of multiple resting-state networks from a single acquisition and providing particular value in non-cooperative, cognitively impaired, aphasic, pediatric, or sedated patients. Evidence indicates that rs-fMRI is most robust for sensorimotor mapping, with reported agreement with tb-fMRI and intraoperative direct electrical stimulation, whereas language mapping remains less consistent and more dependent on analytical methodology. Neither modality replaces intraoperative stimulation, which remains the reference standard. **Conclusions:** Current evidence supports a multimodal presurgical strategy in which tb-fMRI is used first-line in cooperative patients; rs-fMRI is added when task-based mapping is limited or infeasible, and both are interpreted alongside tractography, neuronavigation, and intraoperative mapping.

## 1. Introduction

Accurate functional localization is a central and practical priority in neurosurgery because the ability to identify and preserve motor, language and other eloquent cortical and subcortical regions directly determines the balance between maximal safe resection and postoperative functional outcome in both oncological and epilepsy operations [1,2]. Preservation of function therefore shapes preoperative strategy: mapping is used not merely to annotate anatomy but to define the relationship of a lesion to eloquent regions, support surgical planning, and to help determine when intraoperative mapping is needed to judge resectability [2,3]. Intraoperative direct electrical stimulation (DES) has long provided the most definitive, behaviorally anchored identification of essential cortical sites and remains the reference for determining functional indispensability at the time of surgery [4,5].

In this review, DES is used as an umbrella term for intraoperative electrical stimulation used to interrogate cortical or subcortical function. Direct cortical stimulation (DCS) refers specifically to stimulation of cortical sites, whereas subcortical stimulation refers to stimulation of white-matter pathways during resection. DES/DCS is treated as the main operative reference standard for patient-specific functional localization, but its accuracy depends on stimulation parameters, mapping strategy, surgical exposure, task selection, anesthesia conditions, and patient cooperation.

DES’s strengths include high spatial specificity and immediate behavioral or motor effects, which explains its enduring role as a main clinical reference standard, but its constraints are equally important for preoperative planning. DES is invasive, can require intraoperative stimulation or staged extraoperative monitoring with implanted electrodes, and carries risks such as seizure induction, which limits its practicality as a stand-alone tool for preoperative risk stratification and approach selection [4,6]. For these reasons, noninvasive functional imaging has become an indispensable adjunct to bridge the preoperative and intraoperative worlds: functional magnetic resonance imaging (fMRI) produces individualized maps that can inform risk estimates, guide approach planning, and be integrated with structural MRI and diffusion-based tractography for neuronavigation [2,7]. In routine clinical workflows, task-based fMRI (tb-fMRI) has therefore been adopted widely to localize motor and language domains and to estimate hemispheric language dominance prior to surgery [2].

Despite its clinical uptake, tb-fMRI has fundamental limitations that are especially important in neurosurgical patients. Task paradigms require patient comprehension, attention, and adequate task performance; therefore, these requirements may not be met in patients with aphasia, cognitive impairment, or seizures, producing failed or unreliable maps in some presurgical candidates [8,9]. In addition, blood-oxygen-level-dependent (BOLD) fMRI signals can be attenuated near tumors by neurovascular uncoupling (NVU) and abnormal tumor vasculature, which may cause false-negative activations adjacent to lesions and thereby confound risk assessment [10]. Together, these limitations contribute to meaningful failure rates and reduced confidence in tb-fMRI in some clinical cases, motivating complementary approaches that do not require active task performance, such as resting-state fMRI (rs-fMRI) [7,8].

Rs-fMRI has emerged as a clinically attractive opportunity in this context because it maps intrinsic low frequency BOLD correlations between spatially distributed regions, thereby delineating large scale functional networks, including sensorimotor and language systems, from a single task-free acquisition [11,12]. Clinically, the task-free nature of rs-fMRI makes it feasible in sedated, aphasic, cognitively impaired, or pediatric patients who cannot reliably perform tasks, and it allows acquisition of multiple network maps in a single scan that can be coregistered to anatomy and integrated with diffusion tensor tractography for presurgical planning [7,13]. Importantly, a growing body of translational work reports concordance between rs-fMRI network topography and task activations or DES mappings, supporting the modality’s translational potential, while also highlighting variability related to functional reorganization, lesions, and methodological factors that require careful validation [14,15].

**Figure 1:**
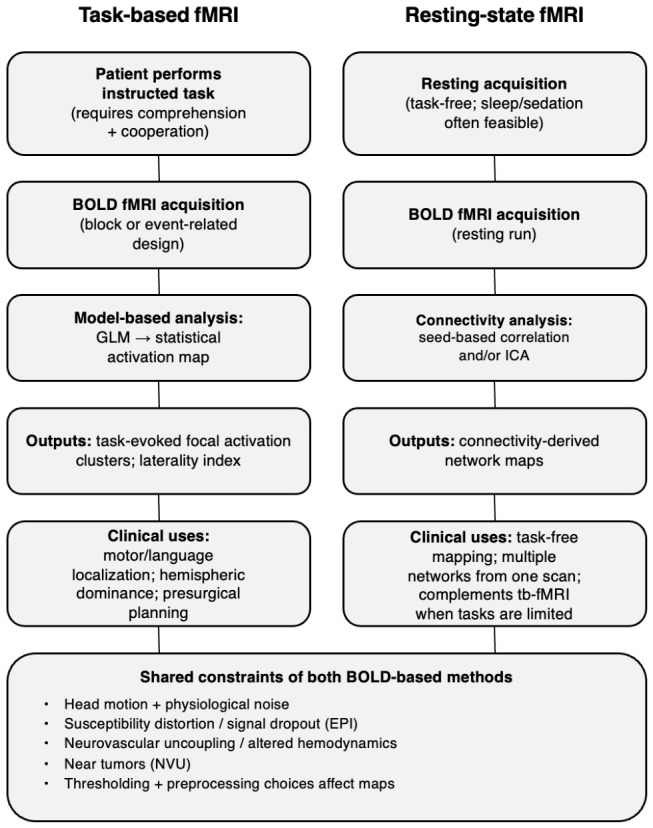
Conceptual workflow comparison of task-based fMRI (tb-fMRI) and resting-state fMRI (rs-fMRI) for presurgical functional mapping, highlighting acquisition requirements, core analysis approaches (GLM vs. connectivity/ICA), map outputs, and shared confounds relevant to neurosurgical planning.

Tb-fMRI acquires BOLD images while the patient performs predefined language, motor, or sensory tasks and identifies cortical sites whose hemodynamic responses temporally covary with the task, thereby localizing putative eloquent regions relevant to the planned operation (Figure 1) [2]. By contrast, rs-fMRI records BOLD signal while the patient is at rest and derives networks by measuring spontaneous temporal correlations among remote regions, with the output being connectivity-based spatial maps of functionally coupled systems (e.g., sensorimotor, language, default mode) that do not require task performance. Both modalities are BOLD-based and therefore subject to vascular and methodological confounds; however, they are complementary: tb-fMRI emphasizes task-evoked specialization while rs-fMRI emphasizes intrinsic network architecture and potential reorganization relevant to individualized surgical risk assessment [11,12].

Motivated by the clinical need to maximize resection while minimizing deficits, by the constraints of intraoperative DES, by the practical limitations of tb-fMRI, as well as by the expanding translational literature on rs-fMRI, this narrative review compares the clinical applicability of rs- versus tb-fMRI across representative neurosurgical populations. It synthesizes evidence on reliability and validation against reference standards, including DES together with task activations, while also appraising strategies, plus practical pipelines for integration of these modalities into presurgical workflows and neuronavigation [7,12,14,16]. These aims address when rs-fMRI should complement tb-fMRI and when it may serve as the primary noninvasive mapping option in patients in whom task-based mapping is infeasible, while emphasizing that connectivity-derived maps require multimodal interpretation and do not by themselves prove functional indispensability.

This review was designed as a clinically oriented narrative synthesis rather than a systematic review or meta-analysis. It was based on a non-systematic literature search of PubMed/MEDLINE, Scopus, Web of Science, and Google Scholar from database inception to March 2026. The review focused on tb-fMRI and rs-fMRI for presurgical functional mapping in neurosurgical populations, including clinical utility, feasibility, validation, limitations, and workflow integration.

Search terms included combinations of “task-based fMRI”, “resting-state fMRI”, “presurgical mapping”, “neurosurgical planning”, “brain tumor”, “glioma”, “epilepsy”, “direct electrical stimulation”, “language mapping”, “motor mapping”, and “neurovascular uncoupling”. Studies were considered relevant when they addressed clinical applications, validation approaches, methodological limitations, or practical workflow considerations for tb-fMRI and rs-fMRI in presurgical functional mapping. Priority was given to studies involving neurosurgical populations, motor or language mapping, comparison with intraoperative electrical stimulation or task-based mapping, neurovascular uncoupling, and multimodal integration with tractography, neuronavigation, and intraoperative mapping. Studies with primarily technical content were included only when they had direct implications for clinical interpretation or workflow implementation.

Compared with prior reviews, the practical contribution and novelty of this article lie in its focus on patient-selection logic and workflow integration: when tb-fMRI should remain the first-line noninvasive approach, when rs-fMRI should be added, when rs-fMRI may serve as the primary noninvasive option because task performance is infeasible, and how both methods should be interpreted within a multimodal neurosurgical framework.

## 2. Clinical Importance of Functional Localization in Neurosurgery

Precise localization of functional cortex and critical white-matter pathways is central to contemporary neurosurgical decision-making as it defines the clinical trade-offs between oncological benefit and preservation of neurological function [17,18]. Greater extent of resection (EOR) has been associated with improved seizure control, delayed progression, and longer survival in gliomas, but proximity to eloquent cortex and subcortical tracts often limits resectability and increases the risk of postoperative deficits. Accordingly, preoperative and intraoperative identification of functionally essential tissue informs the balance between oncological control and functional preservation that guides operative strategy [17,18,19]. Resting-state magnetoencephalography functional connectivity measures have additionally been correlated with early and medium-term neurological morbidity, underscoring that network integrity, not only gross anatomy, predicts outcome [20].

The importance of localization in practice is most evident at three linked decision points: margin choice, corridor or approach selection, and intraoperative EOR. Preoperative functional mapping, together with intraoperative awake mapping, can justify an aggressive, supratotal resection strategy when mapping indicates separable, non-eloquent margins, or it can mandate conservative limits when eloquent tissue is identified adjacent to tumor, thereby changing the planned resection envelope and operative route [17,19]. Cortical and subcortical mapping also accommodates demonstrated neuroplasticity. Repeated mapping has shown that functional representations may shift during resection or between surgeries, and these dynamics can alter the timing and aggressiveness of resection [21,22]. In short, localization translates into concrete intraoperative thresholds for stopping resection, sparing particular gyri or fasciculi, and choosing awake versus asleep techniques [19,23].

Indications for routine functional localization are well established and clinically bounded. The paradigmatic indication is an intrinsic glioma within or adjacent to motor or language cortices and their underlying tracts, where mapping can help define resectability and support maximal safe resection with preservation of function [17,24]. Solitary metastases that adjoin eloquent cortex likewise frequently undergo function-guided resection, including awake mapping, to maximize lesion removal [25,26]. Epilepsy surgery that involves peri-eloquent cortex relies on multimodal functional mapping, including intraoperative adjuncts such as intraoperative MRI and extraoperative electrical stimulation mapping with stereo-electroencephalography for language networks, to distinguish epileptogenic from essential cortex and to plan tailored resections [27,28]. Vascular lesions such as arteriovenous malformations in or near eloquent regions are less frequent but similarly benefit from mapping to reduce iatrogenic deficits and to guide the safest disconnection strategy [29,30].

The clinical use of functional maps is practical and action-oriented. Preoperative maps support individual risk stratification by quantifying location-specific risks and by helping estimate the risk of permanent postoperative deficits [31,32]. Maps are imported into neuronavigation systems for intraoperative localization and trajectory planning, representing core inputs for intraoperative strategy including awake testing, motor-evoked potential monitoring, and subcortical stimulation [32,33]. Tractography provides corridor maps of eloquent white matter pathways and, when tract segments are analyzed, relates subcortical injury or resection to specific neurological deficits, thereby shaping targets for subcortical stimulation and resection strategy [31,34].

FMRI may be positioned within a multimodal apparatus rather than as a standalone arbitrator. DES of the cortex or subcortex and intraoperative electrophysiology remain the main operative reference standards for definitive functional localization and for real-time decision making, but noninvasive preoperative methods including tb-fMRI, resting state connectivity, navigated transcranial magnetic stimulation (nTMS), magnetoencephalography, and diffusion-based tractography provide the roadmap that enables planned, function-preserving resections and interpretation of intraoperative findings [17,35]. Because each modality has strengths and limitations (task dependence, subject cooperation, spatial specificity, intraoperative brain shift), contemporary practice integrates them: preoperative tb- and rs-fMRI plus tractography inform neuronavigation and risk estimates, whereas intraoperative DES with neurophysiological monitoring confirms functional borders and guides the final EOR [17,35,36]. Clinically, this multimodal, map-guided workflow is the mechanism by which functional localization alters management by tailoring the intended EOR and supporting risk counseling for patients with gliomas, metastases, and epilepsy [19,26,27].

## 3. Tb-fMRI in Neurosurgical Planning

Tb-fMRI occupies a pivotal role in presurgical mapping due to its ability to produce spatially-specific, task-driven maps and correlating with behaviorally defined functions such as hand movement and language production [37]. It is relatively widely available, integrated into clinical workflows, and increasingly more often used for presurgical functional mapping in practice [37,38]. Clinically, tb-fMRI generates focal activation maps that localize components of the motor and language networks and provide noninvasive hemispheric lateralization that informs decisions about surgical approach and operative risk [37,39,40]. Task-evoked topography links specific behavioral tasks such as finger tapping or sentence completion to spatial maps of BOLD responses, supporting presurgical planning and interpretation [37,40].

Tb-fMRI generates focal statistical maps of task-related BOLD signal changes that are interpreted as functional loci for motor and language tasks [40,41]. Motor maps typically reveal activations corresponding to the body part engaged by the task, while language paradigms draw out activations in perisylvian regions and additional frontal and temporal sites, depending on the paradigm [40,42]. Tb-fMRI can quantify hemispheric dominance by comparing the extent of language-related activation across homologous regions, often summarized with a laterality index [40,43]. As a noninvasive surrogate for language lateralization, fMRI has been reported to be comparable to the Wada test and is used for preoperative counseling and planning [41,43]. Clinicians use tb-fMRI to localize critical cortical areas adjacent to lesions and to summarize language system laterality when invasive mapping is impractical or undesirable [39,41].

Standard motor paradigms are reproducible tasks such as finger tapping, foot tapping, or sequential finger movements that activate the contralateral sensorimotor cortex [41,42]. In cases of limited active cooperation, such as very young children or patients who cannot comply, passive-motion paradigms performed by an examiner can elicit sensorimotor BOLD responses and facilitate mapping of the sensorimotor cortex [44]. Language mapping employs validated paradigms to probe expressive and receptive components, with tasks such as covert verb generation, sentence completion, and naming selected items based on clinical questions and patient capabilities [40,41]. Because different paradigms recruit different components of the language network and can show limited spatial overlap, task batteries are often used in clinical protocols to improve localization and lateralization [40,45].

Clinically relevant tb-fMRI outputs are provided as statistical activation maps, superimposed on high-resolution anatomical MRI sequences, which can be converted to DICOM and loaded into neuronavigation systems for integration into surgical planning [46]. Surgeons interpret tb-fMRI in relation to tumors or epileptic foci by assessing the spatial relationship between activation foci and lesion margins, informing decisions about surgical planning and selection of patients for intraoperative electrocortical stimulation mapping [41,46]. This view of tb-fMRI maps is useful for preoperative risk stratification but should not replace intraoperative confirmation, as registration errors and intraoperative brain shift can alter fMRI localization during procedures [37,46].

Tb-fMRI’s main strengths are its practical and evidence-based utility: it establishes direct task-to-map correspondence, is deeply integrated into clinical workflows, and has abundant literature supporting its comparison with intraoperative stimulation mapping and usefulness in surgical decision-making, including situations where awake mapping is challenging or not tolerated [37,47]. Test–retest studies demonstrate that tb-fMRI can produce reproducible activation patterns, although reliability varies with factors such as task choice and tumor characteristics [48]. Because concordance with electrocortical stimulation can vary, tb-fMRI is typically evaluated conservatively and, when possible, corroborated intraoperatively [37]. For tumor and epilepsy cases, tb-fMRI is particularly beneficial for presurgical counseling, operative strategy selection, and preparation for adjunctive localization techniques [41,47].

Despite its strengths, tb-fMRI has several limitations that clinicians must consider. The spatial extent of activation is susceptible to factors such as task choice and statistical thresholding, which can lead to over- or underestimation of the extent of functional activation [40,49]. Susceptibility artifacts can cause signal loss and distortion, affecting interpretations of language and semantic sites near lesions [37,38]. Notably, tumor-induced NVU can suppress task-evoked BOLD responses, resulting in false-negative maps and perceived cortical reorganization. This issue has been documented in gliomas and other focal lesions, representing a principal limitation of BOLD-based tb-fMRI [10,37].

In routine neurosurgical practice, tb-fMRI provides task-specific localization that helps plan the surgical approach, estimate proximity-based functional risk to motor and language areas, and anticipate whether intraoperative cortical stimulation mapping during an awake craniotomy is needed [37,46]. In epilepsy surgery, tb-fMRI is generally used to assess language lateralization and, to a minor degree, to determine the intrahemispheric distribution of eloquent cortex [47]. The clinical value of tb-fMRI increases when results are interpreted in a multimodal workflow that integrates high-resolution structural MRI and tractography with intraoperative stimulation mapping and considers NVU through cerebrovascular reactivity (CVR)-based assessment when relevant [10,37,39].

## 4. Limitations and Failure Modes of Task-Based fMRI in Neurosurgical Candidates

Tb-fMRI is widely used for presurgical localization of motor and language cortex. However, its practical value in the neurosurgical setting is constrained by practical limitations and common failure modes that can affect intraoperative strategy and patient outcomes [50,51]. When tb-fMRI fails, it can be non-diagnostic or worse, misleading, producing absent, attenuated, or nonspecific activations that may prompt either unnecessarily conservative resections or inadvertent injury to eloquent cortex [50,52]. Below, we outline key failure modes and their implications for surgical planning.

### 4.1. Aphasia and Language Deficits: Unreliable or Absent Activations and False Negatives

Language paradigms require intact comprehension, task compliance, and preserved articulatory control. In patients with impaired cognition or language these prerequisites may be insufficient, increasing the risk that tb-fMRI language maps are limited or non-diagnostic [50,53]. Comparative work suggests that task paradigms can show variable or unexpectedly sparse activations in brain tumor patients, whereas resting-state approaches can provide language network maps without task performance and can be useful when tb-fMRI is limited [50,54]. Because preoperative language localization informs surgical planning and may guide intraoperative mapping, an absent or very focal tb-fMRI map in a patient with limited task compliance should be interpreted as potentially non-diagnostic rather than as evidence of absent function. In such cases, additional modalities such as DCS or rs-fMRI are often needed [50,53,54].

### 4.2. Cognitive Impairment, Fatigue, Anxiety, and Pain: Performance Variability, Noncompliance, and Motion

Neurocognitive deficits in brain tumor patients can limit cooperation with tb-fMRI paradigms, which can make consistent task performance difficult [55]. Head motion is a key confound in resting-state functional connectivity analyses and is typically addressed with motion correction and frame censoring during preprocessing [56]. Because task participation can be challenging, rs-fMRI has been proposed as a less demanding alternative for presurgical functional mapping, and resting-state connectivity measures may support pre-treatment planning and patient counseling [55,56].

### 4.3. Motor Impairment and Paresis: Inability to Execute Paradigms and Compromised Mapping

Motor tb-fMRI presumes the ability to perform simple movements, for example, finger tapping, but paresis or paralysis may prevent execution and therefore prevent reliable motor mapping [51,57]. In stroke and severely impaired patients, tb-fMRI may be confounded by changes in performance, and rs-fMRI may help characterize residual or reorganized motor networks when voluntary movement is not possible [57,58]. Because tb-fMRI localization can be challenging in patients with paresis or paralysis, absent motor activation in a weak or plegic limb should be interpreted cautiously, and complementary mapping methods such as nTMS or intraoperative electrical stimulation may be considered to reduce the risk of postoperative motor deficit [51].

### 4.4. Pediatric and Other Non-Cooperative Patients: Developmental Limits and Sedation Constraints

Children and non-cooperative adults are frequently unable to understand or sustain task demands. In pediatric epilepsy and brain tumor cohorts some patients are unable to participate in tb-fMRI language paradigms due to young age, cognitive impairment, or poor task performance. Moreover, sedation or general anesthesia may be used for young children or uncooperative patients, which makes task paradigms unfeasible, while rs-fMRI can be performed without active task performance, including during sleep or anesthesia. When task paradigms are not feasible or are limited, alternative strategies such as rs-fMRI and, when clinically indicated, invasive mapping approaches such as Wada testing or cortical stimulation mapping may be needed for preoperative planning [50,59].

### 4.5. Tumor Physiology and Neurovascular Confounds: Attenuated and Displaced BOLD Responses

One important physiological limitation for neurosurgeons is tumor-related NVU and altered hemodynamics in peritumoral tissue. Tumors can induce vascular and metabolic changes that attenuate or abolish the BOLD response, contributing to false-negative tb-fMRI maps [42,52]. Hemodynamic calibration in tumor subjects shows substantial voxel-wise variability in task-induced BOLD responses, which along with a breath-hold measure can change the apparent extent of activation, with published comparisons with DCS reporting variable sensitivity and specificity for motor fMRI. BOLD magnitude is also modulated by non-neural factors such as cortical thickness, depth, and macrovasculature, which can confound interpretation [52,60]. As these effects can cause tb-fMRI to underrepresent eloquent cortex near lesions, tb-fMRI alone should be interpreted cautiously when planning resection around tumors or other lesions with altered vascular reactivity [42,52].

### 4.6. Practical Implication: tb-fMRI Fails Most Often in the Patients Who Need Accurate Mapping the Most

Collectively, these failure modes indicate that tb-fMRI may be non-diagnostic or even misleading in the highest-risk patient populations, including individuals with cognitive or language impairments who are unable to adequately perform paradigms, patients with motor deficits, pediatric patients who cannot reliably cooperate, and those with tumors or peritumoral pathology in whom altered hemodynamics may attenuate or distort BOLD activation. Paradoxically, these are precisely the patients for whom accurate functional localization is most critical. In such contexts, rs-fMRI or intraoperative electrical stimulation mapping may therefore be required as complementary or alternative approaches [50,51,52].

### 4.7. List of Key Failure Modes and Surgical Consequences

- Language impairment: absent or unreliable tb-fMRI activations can limit identification of eloquent language cortex for surgical planning; therefore, alternative approaches such as rs-fMRI or intraoperative mapping with DCS may be warranted [50,54].
- Cognitive impairment or reduced ability to cooperate: inconsistent task performance and head motion can produce limited or non-diagnostic tb-fMRI maps, increasing reliance on anatomical landmarks and invasive mapping. In such cases, complementary techniques like rs-fMRI or additional neurophysiological testing may be considered [50,54].
- Motor impairment or paresis: inability to execute motor tasks can compromise task-based mapping of sensorimotor function, which may necessitate the use of rs-fMRI or complementary mapping methods such as nTMS or intraoperative stimulation [51,57].
- Pediatric, noncooperative, and sedated patients: developmental limitations, sedation or anesthesia can make it difficult to remain still and perform timed tasks, which can render tb-fMRI impractical. In these situations, rs-fMRI and, when needed, intraoperative mapping may be considered [42,59].
- Tumor physiology, including altered hemodynamics: neurovascular changes can attenuate or distort task-evoked BOLD responses, leading to false negative findings or spatially shifted activations that may misinform resection planning. Calibration with CVR measures, such as breath-hold mapping, and awareness of non-neural vascular or anatomical influences on BOLD magnitude can help interpret these maps [52,60].

Clinically, recognizing these failure modes and their potential operative consequences is essential for the realistic interpretation of tb-fMRI and for guiding the selection of complementary approaches, such as rs-fMRI and DCS, when task-based maps prove unreliable (Table 1).

## 5. Resting-State fMRI for Presurgical Mapping

Rs-fMRI is based on the observation that spontaneous, low-frequency (less than 0.1 Hz) fluctuations of the BOLD signal during rest are temporally correlated across spatially distributed brain regions, indicating functional connectivity between anatomically remote areas [61,62,63]. These correlations form reproducible resting-state networks (RSNs) that reflect intrinsic large-scale neural networks and can be used for task-free functional localization in presurgical planning [12,64]. For neurosurgical planning, rs-fMRI can derive spatial maps of multiple distributed networks from a single resting BOLD acquisition, helping identify eloquent cortex and connected circuits that may be at risk during resection [12,16].

In presurgical practice, particularly relevant RSNs include the sensorimotor network (SMN), which comprises primary motor and somatosensory cortices, language networks, which involve frontal and temporal perisylvian nodes, and higher-order networks such as the default mode network (DMN), which has been linked to episodic memory and related cognitive performance [3,65]. Clinical studies in patients undergoing tumor surgery have shown strong spatial agreement between rs-fMRI-derived sensorimotor and language networks, and intraoperative DES findings, supporting the clinical validity of rs-fMRI for eloquent cortex mapping [14,65]. Rs-fMRI network metrics have also been used to quantify perioperative connectivity changes and relate them to postoperative outcomes in neurosurgical populations [66,67]. Additionally, automated rs-fMRI pipelines can estimate higher-order networks, including the DMN and frontoparietal networks, which are difficult to probe intraoperatively and may be relevant to postoperative cognitive outcomes [3,14,65].

Clinically, rs-fMRI yields spatial network maps rather than focal task-evoked activation clusters from tb-fMRI, with outputs typically presented as seed-derived correlation overlays or as spatial independent component maps representing whole-brain RSNs from a single resting run [12,16,50]. This methodology enables the concurrent extraction of multiple clinically pertinent networks (motor, language, visual, DMN, attention) from a single resting run, and the resulting maps can be overlaid on co-registered anatomical MRI for presurgical planning, similarly to tb-fMRI [12,16].

From an operational standpoint, rs-fMRI can be relatively efficient to implement: in presurgical protocols it is often acquired as one or two short resting BOLD runs, and some clinical workflows explicitly favor two ~6 min runs (≈12 min total) to preserve enough usable data after motion-related frame loss and other exclusions [12,64]. Instructions are typically minimal (e.g., remaining still with eyes closed), which further reduces setup complexity. Because rs-fMRI does not require active task performance, it is especially useful when task paradigms are not feasible, such as in young children, sedated patients, or individuals with cognitive/neurological deficits. Furthermore, it can provide broad network information with comparatively limited demands on patient cooperation [64,68,69]. Collectively, these features can make rs-fMRI easier to integrate into presurgical imaging workflows, although acquisition details and run duration may vary by site and clinical constraints.

In clinical analysis, two primary strategies dominate: hypothesis-driven seed-based correlation mapping and data-driven independent component analysis (ICA). Each has distinct advantages and limitations that should influence method selection [12]. Seed-based approaches involve placing a small region of interest, such as hand motor or supplementary motor area seeds, and computing voxelwise correlation maps. This method is intuitive and effective for focal targets, but it depends heavily on accurate region of interest placement and can be compromised when brain shifts or tumor-related distortion precludes reliable seed positioning [70]. Conversely, independent component analysis decomposes the resting-state BOLD time series into statistically independent spatial components, which can reveal multiple RSNs from one acquisition and support denoising by identifying noise components for removal [13,71]. However, the component representing the functional system of interest may be fragmented across several components otherwise not straightforward to identify; therefore, visual inspection or manual selection is often required unless automated template matching or classifiers are used [13,16].

Because presurgical decisions depend on spatial precision, rs-fMRI processing can incorporate targeted artifact mitigation approaches, including distortion correction strategies, multiecho acquisitions with ICA-based separation of BOLD and non-BOLD components, and the use of spin-echo echo-planar imaging to improve connectivity mapping in susceptibility-affected regions. Additional corrections may be needed when vascular transit delays are expected to bias connectivity estimates [72,73,74]. Awareness of physiological confounds such as slow respiration-related fluctuations and clear documentation of preprocessing decisions such as global signal regression are essential, as these choices can substantially influence connectivity map appearance along with interpretation [73,75,76].

A practical workflow for a neurosurgical team utilizing rs-fMRI involves:

- Ordering rs-fMRI alongside routine preoperative structural imaging, providing minimal, standardized patient instructions, and acquiring roughly 8 to 12 min of resting-state data in one or two runs [12,77].
- Preprocessing with motion correction, distortion correction, and a denoising strategy such as ICA-based cleaning with FIX [71].
- Selecting between seed-based correlation analysis or independent component analysis-based approaches depending on the clinical question and producing network overlays matched to the anatomical T1-weighted volume [12,77].
- Co-registering rs-fMRI network maps to individual anatomy and creating overlays for presurgical planning and multidisciplinary review, while documenting limitations and, where feasible, reviewing results alongside tb-fMRI, tractography, and intraoperative mapping [14,65,66].
- Validating rs-fMRI network localization against intraoperative DES when possible and applying structured interpretation and reporting criteria with institution-specific thresholds as experience accumulates [14,67].

When implemented systematically, rs-fMRI provides clinically actionable whole-brain network information that complements tb-fMRI, with the ability to be incorporated into presurgical planning and intraoperative guidance to help tailor tumor resections and mitigate surgical risks [14,65,66].

## 6. Clinical Evidence and Validation

Validation of rs-fMRI for presurgical functional mapping relies on clinical evidence examining concordance with tb-fMRI, concordance with intraoperative DCS or subcortical stimulation (DES), reliability, and particular performance contexts (motor versus language and perilesional caveats).

Reviews and comparative studies indicate a consistent pattern: rs-fMRI provides reliable localization of sensorimotor systems and whole-brain coverage that is useful in patients unable to perform tasks, whereas language mapping is more variable, and tumor-proximate neurovascular abnormalities can further complicate interpretation. Rs-fMRI is, therefore, promising as an adjunct, but not as a replacement for intraoperative stimulation mapping [12,64,78].

### 6.1. Motor Network Mapping

#### 6.1.1. Concordance with tb-fMRI Localization

Multiple studies and reviews report substantial spatial overlap between motor networks identified with rs-fMRI and tb-fMRI, with clinical series reporting concordance with intraoperative stimulation mapping [12,78]. This concordance likely reflects the consistent anatomical localization of the sensorimotor system to the precentral and postcentral gyri and the temporally synchronous spontaneous activity captured by rs-fMRI, which facilitates identification by automated data-driven or template-based rs-fMRI methods [16,79].

#### 6.1.2. Concordance with DCS/DES

Validation against intraoperative DES, which is considered the main clinical reference standard for identifying indispensable cortex during awake craniotomy, is a direct test of clinical utility [14]. Recent small series and tool validation reports have shown that rs-fMRI sensorimotor maps often localize near sites where cortical DES produces motor or sensorimotor interference, with many stimulation-positive points lying within about 1 cm of the corresponding network. These studies also highlight that reported concordance depends on preprocessing and network selection choices, as well as on how spatial agreement is quantified, underscoring that further validation in larger, multisite cohorts is still needed [14,16].

#### 6.1.3. Test–Retest Reliability Themes

The SMN is among the most reliably detected resting networks across imaging sessions, yielding acceptable test–retest reproducibility for presurgical mapping [79,80]. Reliability can be improved by standardized acquisitions, motion mitigation, and pipeline choices such as ICA-based or classifier-based network extraction, but it is reduced by head motion, physiological noise, and peritumoral hemodynamic confounds. These technical factors are well documented and require routine QC in clinical workflows [10,64,78]. Longitudinal studies also show that sensorimotor connectivity can recover or reorganize after surgery, supporting the clinical sensitivity of SMN measures for serial imaging [81].

### 6.2. Language Network Mapping

#### 6.2.1. Distributed Anatomy and Variability

Language mapping presents greater challenges than motor mapping because the language system involves many regions and shows substantial interindividual variability in the localization of key nodes. Rs-fMRI can reflect these distributed systems, but it often yields spatially wider and less focal maps than task activation [78]. Consequently, network parcellation and analysis choices, including seed placement or selection of an independent component, substantially influence the apparent topography of language-related resting-state networks [64,78].

#### 6.2.2. Evidence for Lateralization Concordance

In brain tumor patients, rs-fMRI connectivity can show close spatial agreement with tb-fMRI and, in many cases, with DES for language network localization [14,78]. Nevertheless, concordance for language mapping is generally lower and more variable than for motor mapping [78]. Tumor-related neurovascular coupling disruption can compromise BOLD-based measures and complicate interpretation of language laterality and localization near the lesion [43,82].

#### 6.2.3. Practical Interpretation: Adjunctive, Not Definitive

The collective literature supports a practical approach: rs-fMRI language maps provide hypothesis generating information about the distributed architecture of language systems that is valuable for surgical planning, for example, by identifying candidate language network regions and potential contralateral reorganization. However, they do not remove the need for focal intraoperative DES to resolve critical, patient-specific cortical sites that influence postoperative language outcome [10,14,16]. In practice, many teams use rs-fMRI to inform surgical strategy and to prioritize regions for awake mapping rather than to replace DES entirely [12,78].

### 6.3. Tumor Patients: Perilesional Issues

#### 6.3.1. Mechanisms and Manifestations of Distortion

Gliomas induce profound alterations in local hemodynamics, vascular architecture, and in neurovascular coupling, leading to attenuation, displacement, or distortion of the BOLD signal underlying both task-based and resting-state fMRI measures [82,83,84]. These tumor-related changes, which are referred to as NVU, have been demonstrated in experimental models as well as in human imaging studies and are frequently invoked to account for false-negative fMRI activation maps in regions adjacent to tumors [10,82].

#### 6.3.2. Rs-fMRI Resilience and Limits

Several authors have reported that rs-fMRI can nonetheless provide clinically useful network maps in cases where task performance or task activation magnitude is poor, as it does not require active task execution [10,16]. Yet studies in glioma patients show reduced or asymmetrical sensorimotor resting-state connectivity related to tumor characteristics, including position, grade, and perfusion, suggesting that rs-fMRI is not uniformly immune to tumor-related NVU [10,84]. Importantly, rs-fMRI reduces dependence on active task performance but does not overcome neurovascular uncoupling, because both tb-fMRI and rs-fMRI rely on BOLD signal changes that can be altered by tumor-related vascular changes, edema, mass effect, abnormal perfusion, and perilesional hemodynamic disturbances. The appropriate stance is therefore cautious optimism: rs-fMRI is promising for mapping in the presence of perilesional pathology, but the problem of NVU is not solved, and reliable clinical use requires explicit detection and mitigation of vascular confounds [10].

#### 6.3.3. Mitigation Strategies

Parallel hemodynamic assessments such as CVR mapping with BOLD using breath-hold or CO_2_ paradigms, arterial spin labeling-based techniques, and multimodal integration strategies that combine rs-fMRI with diffusion tractography and perfusion measures have been proposed to identify NVU and to increase confidence in rs-fMRI-derived maps [10,85]. When NVU is suspected, intraoperative cortical stimulation mapping remains a critical adjunct [10].

### 6.4. Patients in Whom rs-fMRI Adds the Most Value

#### 6.4.1. Clinical Groups with Few Alternatives

Rs-fMRI is particularly valuable when task performance is compromised or impractical. Patients with aphasia, children, those requiring sedation, or cognitively impaired patients benefit from a task-free acquisition capable of generating multiple functional maps from a single scan. In these settings, rs-fMRI provides a feasible whole-brain functional survey that can be integrated into surgical planning and neuronavigation, while also informing intraoperative mapping strategies and surgical approach [14,64].

#### 6.4.2. Coverage and Feasibility

The ability of rs-fMRI to generate motor, language, visual, and other network maps from a short acquisition, typically about 6 to 12 min, confers a practical advantage for comprehensive preoperative coverage, but centers must implement standardized acquisition, preprocessing, and QC workflows to ensure clinical reliability [16,64]. Motion control, physiological monitoring, and rapid expert review are operational prerequisites for safe clinical adoption [64,78].

### 6.5. Synthesis and Practical Recommendations

Available evidence indicates that rs-fMRI performs most robustly for sensorimotor localization, where concordance with tb-fMRI and DES is high, whereas language network localization is more variable, particularly in posterior language regions [14,78]. Tumor-related NVU remains a major biological confound, and although CVR mapping can assist in detecting and mitigating NVU, it cannot fully eliminate the associated uncertainty [10]. Accordingly, current data support the cautious integration of rs-fMRI into the presurgical armamentarium, particularly for patients unable to perform tasks or when broad network coverage is desirable, while maintaining intraoperative stimulation as the definitive method for identifying indispensable cortex (Table 2) [10,14,78].

## 7. Integration into Practice

### 7.1. Purpose and Overview

The goal of a practical workflow is to convert multimodal preoperative imaging into prioritized, actionable intraoperative guidance while acknowledging the limits of preoperative maps, including brain shift, and recognizing that intraoperative electrostimulation remains the reference standard for functional localization [86,87]. Established methods for multimodal image registration and fusion provide the technical basis for such workflows, but the limitations of preoperative datasets mean that fused anatomical overlays should be treated as navigational aids rather than definitive intraoperative truth [86,88]. Integrating tb- and rs-fMRI into this pipeline requires clear criteria for selecting each approach, attention to the technically demanding postprocessing for rs-fMRI, and interpretation of functional maps alongside structural MRI and diffusion-based tractography, with intraoperative electrostimulation remaining the reference standard for functional localization [87].

### 7.2. Preoperative Workflow: When to Obtain tb-fMRI, When to Add rs-fMRI, and When rs-fMRI May Be Primary

For cooperative adults and older children who can perform reproducible motor and language tasks, tb-fMRI is commonly used as a first-line cortical localization modality, because it yields task-evoked focal activations that can be related to operative anatomy and used for preoperative planning [89,90]. Diffusion imaging for tractography should be acquired concurrently so that subcortical relationships, for example the corticospinal tract and arcuate fasciculus, are available during planning [90,91]. Rs-fMRI should be added when task compliance is poor, including young children, cognitively impaired patients, or patients with severe aphasia [89]. Seed-based and network-level rs-fMRI analyses have been used to localize SMN and language related networks and to complement tb-fMRI, particularly when task performance is unreliable [89,91]. In patients who are non-cooperative or cannot perform tasks, including children, anesthetized or heavily sedated patients, rs-fMRI can serve as a primary paradigm-free mapping modality to provide a network context for planning [89]. Validation with other modalities, including diffusion tractography, nTMS, and intraoperative stimulation, will remain necessary [89,91].

### 7.3. Practical Preoperative Acquisition and Fusion Steps

A concise and reproducible acquisition and QC strategy facilitates reliable downstream integration. This approach may include acquisition of high-resolution structural MRI (T1-weighted, with contrast when indicated) for neuronavigation co-registration, routine anatomical sequences (T2/FLAIR) to delineate lesion margins, diffusion sequences optimized for tractography, tb-fMRI task runs (motor and/or language paradigms appropriate to the lesion), and one or more rs-fMRI runs analyzed using established pipelines (seed-based or independent component analysis, according to local expertise). Multimodal image registration and image fusion pipelines are recommended to minimize alignment errors and to generate fused volumes compatible with neuronavigation systems [86,88]. Registration and fusion quality should be evaluated, and any limitations that may affect navigation should be documented, including the potential need for intraoperative updates when deformation or brain shift is anticipated [88,91].

### 7.4. Neuronavigation Integration and Practical Utility for Approach Planning

Following QC, the structural MRI should be imported into the neuronavigation environment together with overlay volumes, such as rs-fMRI network nodes and tractography datasets, using rigid registration tools and, where available, elastic registration methods [86,91]. Functional activations and networks can be displayed as adjustable-transparency overlays on 3D reconstructions, enabling visualization of cortical regions of interest in relation to planned craniotomy margins and surgical approach corridors [88,92]. Fused tractography visualizations should be presented concurrently to evaluate the spatial relationship between these cortical regions and critical white matter pathways, supporting approach planning and selection of cortical entry points [91]. In practical terms, this integrated visualization framework facilitates definition of a safe surgical trajectory that preserves both cortical and subcortical pathways while enabling maximal safe tumor resection [91].

### 7.5. Multimodal Strategy: Combining fMRI with Diffusion Tensor Imaging/Tractography and Intraoperative Mapping

When using tractography to support risk assessment for subcortical resection, preoperative tractography of the corticospinal tract and language tracts, such as the arcuate fasciculus, should be generated and integrated into the neuronavigation dataset to visualize their relationship to the lesion and to help plan approaches that avoid critical pathways [90,93]. Important caveats are diffusion tensor imaging’s (DTI’s) susceptibility to distortion and interalgorithm variability. Therefore, tractography must be interpreted in context and compared with other modalities and intraoperative findings rather than treated as definitive [94,95]. In multimodal neuronavigation, preoperative fMRI and DTI tractography can be used to project cortical regions and nearby white matter pathways in the operative view and to guide intraoperative electrophysiological monitoring and stimulation when indicated. NTMS targeting is similarly dependent on accurate neuronavigation and registration [96,97]. Preoperative determination of 3D relationships between lesions and eloquent structures can enable a smaller, tailored craniotomy and corticectomy close to the lesion, with intraoperative MRI and monitoring used to correct for brain shift and to assess resection [96].

### 7.6. Brain Shift, Intraoperative Updating, and the Continued Role of Direct Mapping

Preoperative maps cannot fully account for intraoperative brain shift [91,98]. Intraoperative MRI and interval ultrasound can be used to update neuronavigation and mitigate brain shift during resection [98]. Intraoperative CT with brain shift correction using elastic or other non-rigid registration can also update preoperative functional and tractography data intraoperatively [91]. Even with these updates, functional localization still needs confirmation with DES and neurophysiological monitoring. Importantly, preoperative rs-fMRI network maps and tractography can still help interpret displaced anatomy and help decide where to prioritize intraoperative mapping, but they do not replace stimulation-based functional confirmation [87,91]. Therefore the practical workflow is as follows:

- Acquire tb- and/or rs-fMRI with DTI preoperatively, followed by fusing them into neuronavigation to plan the approach and prioritize targets [87,91].
- Use intraoperative imaging to update the fused dataset when available [91,98].
- Confirm and refine functional boundaries with DES and intraoperative neurophysiological monitoring, including cortical or subcortical mapping when feasible, as the definitive guide to resection boundaries [87,91].

### 7.7. Final Note and Recommendations

For routine neurosurgical planning, a complementary strategy in which tb-fMRI serves as the first-line option for cooperative patients may be recommended, as it enables task-specific localization and may reveal atypical functional reorganization. Rs-fMRI can be incorporated when tb-fMRI acquisition is incomplete or when task performance is not feasible [87,89,99]. Functional maps derived from these approaches may be interpreted in conjunction with DTI tractography to delineate critical white matter pathways. Final surgical decisions should ultimately rely on DES mapping, supplemented, when available, by imaging modalities such as intraoperative MRI [87,99].

## 8. Controversies and Barriers to Routine Adoption

Rs-fMRI has compelling logistical advantages for presurgical mapping, including task-free acquisition and applicability when task performance is impractical; however, these practicalities coexist with well-described susceptibility to confounds and dependence on analytical choices [100,101]. The principal barriers cluster around:

- Motion and physiological noise (most problematic precisely in the pediatric, uncooperative, or cognitively impaired populations for whom rs-fMRI is most attractive) [100,102].
- Extensive preprocessing and analytical variability, with limited consensus about which steps to include and in what order [103].
- Limits of mechanistic specificity, because statistical connectivity measures capture associations that can be consistent with multiple causal explanations [104].
- Heterogeneity in the performance and trade-offs of confound-regression strategies across datasets and benchmarks, motivating standardized and transparently reported workflows [101,103].

### 8.1. Motion and Physiological Noise

Head motion produces spatially structured, temporally complex artifacts that bias connectivity measures even for very small displacements, and this problem is accentuated in children, patient populations, and others who cannot reliably perform tasks, which are groups for which rs-fMRI is often proposed as an alternative to task paradigms [100,105]. Cardiac and respiratory cycles, including respiration-driven head motion, introduce structured variance that can be difficult to disambiguate from neuronal BOLD fluctuations [102,106]. Although a range of correction methods (scrubbing, nuisance regression, component-based denoising, and emerging machine-learning approaches) can reduce artifact variance, none removes motion and physiological confounds completely, and residual artifacts can still bias group comparisons in precisely the groups where rs-fMRI is most appealing [107,108].

### 8.2. Preprocessing Variability and Clinical Consequences

The absence of an accepted, single preprocessing pipeline is not merely an academic nuisance: the order and choice of steps (motion regression, temporal filtering, component-based correction, scrubbing, and global signal operations) are non-commutative and can reintroduce or reshape artifacts, producing divergent network topographies from identical raw data [103]. Controversies such as global signal regression illustrate the clinical stakes because it can reduce global artifacts but may also introduce anti-correlations and alter connectivity maps, affecting map interpretability [76]. Benchmarking studies show that different confound-regression and censoring strategies yield markedly different results, with clear trade-offs between residual motion dependence, distance-dependent artifact, network identifiability, and degrees of freedom retained [101].

### 8.3. Specificity Limits: Connectivity Is Not Causality

Functional connectivity metrics quantify statistical dependence, but they do not by themselves identify directed, mechanistic, or functionally critical nodes of a network [104,109]. Methods that attempt causal or directed inferences, such as Granger-type autoregressive approaches or model-based Dynamic Causal Modeling, require explicit assumptions about temporal structure, hemodynamics, spatial aggregation, and noise. They can be sensitive to limited temporal resolution and model misspecification in BOLD data [110,111]. In the presurgical setting, these interpretive limits and analysis choices can yield maps that overestimate or underestimate the extent of an eloquent network, which has practical implications for resection planning [42,103].

### 8.4. Inter-Center Variability and the Need for Validated Clinical Pipelines

Heterogeneity in acquisition and analysis across centers, including differences in pulse sequences, sampling rates, physiological monitoring, preprocessing strategies, and QC criteria, can introduce site-related batch effects that undermine reproducibility and limit the transportability of rs-fMRI maps into routine surgical practice [103,112]. Addressing these challenges requires clinically validated and publicly described pipelines with prespecified QC and exclusion rules, together with direct validation against perturbational benchmarks such as electrical stimulation and outcome-based concordance analyses to estimate sensitivity and specificity in relevant patient populations [42,113].

### 8.5. Clinical Positioning and Practical Recommendations

For current practice, the most defensible position is that rs-fMRI should be regarded as a complementary tool whose interpretation depends on careful control of head motion and other nonneuronal confounds, as well as on transparent reporting of preprocessing choices [101,103,107]. For clinical translation and to meet the expectations of both clinical and regulatory review, investigators should explicitly quantify motion and define clear QC and exclusion criteria. In addition, confound regression strategies and the ordering of preprocessing steps should be transparently documented, or alternatively implemented within unified modeling frameworks, to prevent inadvertent reintroduction of artifacts, an issue that has been repeatedly emphasized in prior methodological work [101,103,107]. These practices help reduce spurious connectivity findings and improve comparability across studies [101,103,107]. Until standardized workflows that reliably prevent artifact reintroduction are consistently applied, rs-fMRI connectivity estimates should be interpreted with caution [103].

## 9. Future Directions

The next translational phase for presurgical fMRI may be organized around four priorities: hybrid task-based and resting-state paradigms; integration with diffusion tractography to support presurgical risk estimation; automated processing and clinical workflow integration; and harmonized acquisition and reporting standards that can be evaluated against intraoperative mapping and clinical outcomes [7,114,115]. Evidence reviews and single-center implementation reports consistently highlight these areas as key drivers of clinical utility and broader generalizability [7,64,114].

Hybrid task-based plus resting-state paradigms represent a practical near-term strategy, as each modality contributes complementary strengths and enables cross-validation for presurgical localization. Comparative studies report moderate concordance, substantial subject-level variability, and differences in spatial extent between task-based- and resting-state-derived maps. These findings support the use of resting-state methods to supplement or validate task-based results rather than to substitute for them uncritically [78,116].

Practical hybrid workflows include using task activations to guide resting-state seed placement when available, as well as relying on resting-state networks when tb-fMRI is not feasible because of poor compliance or when BOLD mapping may be affected by altered neurovascular coupling [53,78]. At the same time, resting-state results remain sensitive to thresholding and analytical choices, and task-derived seeds may overestimate task-free reproducibility. Accordingly, cross-modal validation and transparent reporting of methods remain essential [116,117].

Connectomics provides a conceptual and analytical framework for shifting clinical practice from focal localization toward network-informed, quantitative risk assessment [118]. The functional connectome has been proposed and piloted as a clinical biomarker space for predicting behavior, overall survival, and cognitive reserve. In addition, connectome-based predictive modeling can generate individualized prognostic metrics that may support treatment planning [118,119,120]. Recent advances in effective and dynamic connectivity, including regression dynamic causal modeling and dynamic functional connectivity fingerprints, enable directed and time-resolved characterization of tumor-related network alterations. These approaches may provide information about cognitive status beyond what can be inferred from static focal maps alone [121,122]. Achieving clinical applicability will require patient-specific parcellation strategies and atlases to support accurate, individualized connectome construction [123,124].

Artificial intelligence and automation are required to scale task-based and resting-state fusion, connectome extraction, and outcome prediction into routine clinical practice. Large single-institution translational studies have demonstrated that automated pipelines can be integrated into clinical workflows, although postprocessing and QC review remain resource intensive [7]. Recent machine learning and deep learning approaches, including graph convolutional models, have demonstrated feasibility for automated localization of epileptogenic zones from rs-fMRI connectivity and for automated classification of functional brain networks using transfer learning. However, these methods have largely been evaluated in limited single-center cohorts, underscoring the need for broader validation across scanners, institutions, and pathologies [125,126]. Importantly, translational tools intended for clinical use should incorporate explicit QC and account for NVU, as NVU can suppress task-evoked BOLD activation and may result in misleading functional maps if uncorrected [7,127].

Standardization of acquisition and analysis, together with consistent QC practices, remains essential, as methodological variability leads to widely discrepant performance estimates across studies and complicates multicenter comparisons [114,128]. Key priorities include embedding physiological QC metrics within preprocessing pipelines, adopting explicit atlas and seed conventions to improve cross-site comparability, and conducting benchmarking studies that directly compare task-based and rs-fMRI mapping against intraoperative DCS [53,114,128]. Professional societies and journals can further accelerate clinical adoption by promoting consensus standards for scanning procedures, task administration, and statistical postprocessing in presurgical fMRI [115].

Outcome prediction that anticipates postoperative deficits, recovery trajectories, and plasticity potential represents the ultimate clinical metric for justifying routine use of combined task-based and resting-state approaches. Retrospective studies suggest feasibility, as tb-fMRI metrics such as lesion-to-activation distance and language lateralization have been associated with postoperative language deficits following tumor resection. In addition, combined fMRI, rs-fMRI, and diffusion imaging measures have shown potential for predicting postsurgical cognitive change in temporal lobe surgery. However, this literature also highlights the need for larger prospective datasets and standardized outcome definitions [129,130]. Multimodal models that integrate tb-fMRI or rs-fMRI with diffusion tractography demonstrate additive value for characterizing functional networks and for outcome prediction, making them priority targets for multicenter validation efforts [130]. In the stroke literature, rs-fMRI connectivity measures, including motor network connectivity that is often considered alongside structural pathway measures, are likewise being evaluated for prognostic utility, with repeated calls for larger and more standardized studies [131]. Candidate plasticity markers, such as reduced DMN deactivation in glioma and dynamic resting-state signatures of executive networks, should be assessed prospectively as predictors of postoperative cognitive deficits and recovery trajectories [132,133].

To translate these ambitions into routine practice, four clinical-translation milestones may be proposed:

- Standardize the workflow: publish and promote adoption of an open, validated presurgical fMRI pipeline that incorporates NVU and CVR QC, together with clearly reported test–retest reliability metrics [68,134].
- Benchmark against ground truth and outcomes: conduct prospective, multicenter benchmarking studies that compare hybrid task-based and resting-state outputs against intraoperative mapping and standardized functional outcomes [78,114].
- Make decision support trustworthy: develop and independently validate explainable artificial intelligence tools for network localization and outcome prediction, with regulatory pathways and reporting standards explicitly defined [125,135].
- Prove clinical impact at the bedside: integrate connectome-derived risk metrics into preoperative counseling and into prospective trials designed to test their effects on EOR and patient-centered outcomes [118,119,130].

Achieving these milestones would advance preoperative fMRI beyond modality-specific activation maps toward actionable, network-informed decision support for neurosurgical planning.

## 10. Conclusions

Tb-fMRI remains a widely used, noninvasive tool for presurgical localization of eloquent cortex, but its practical utility is frequently constrained by patient cooperation and by physiological and methodological confounds [42,136]. NVU in perilesional tissue can attenuate task-evoked BOLD responses, and head motion, impaired task performance, and limited test–retest reliability can further degrade signal quality and clinical interpretability in many surgical candidates [10,134,136].

Rs-fMRI complements tb-fMRI by mapping intrinsic functional connectivity without task performance, allowing extraction of multiple RSNs from a single acquisition and enabling application in pediatric, cognitively impaired, or otherwise uncooperative patients [64,136]. Comparative studies demonstrate higher concordance between rs-fMRI and tb-fMRI for core SMNs, which are among the most robust RSNs for presurgical mapping, and lower concordance for language mapping, which depends on the analytical approach [8,136]. These findings support the clinical use of rs-fMRI as a task-free adjunct, particularly for motor mapping and in cases where tb-fMRI is infeasible [7].

However, rs-fMRI is not yet a universal replacement. Lesion-related factors such as NVU and anatomic distortion can complicate BOLD-based localization, and corroboration with other modalities, including intraoperative stimulation when feasible, remains important before fMRI findings alone are used to alter surgical strategy [46,137]. The available evidence therefore supports integration of rs-fMRI within multimodal presurgical workflows alongside tb-fMRI, diffusion tractography, and, when available, direct cortical or subcortical stimulation, in order to maximize localization accuracy and patient safety [7,138].

This review has several limitations. It is a narrative review and not a formal systematic review or meta-analysis; therefore, study selection was not based on a prospectively registered protocol and quantitative pooling was not performed. The available evidence is heterogeneous with respect to patient populations, clinical indications, acquisition protocols, preprocessing pipelines, network-identification methods, reference standards, and outcome measures. In particular, rs-fMRI studies vary substantially in motion handling, seed or component selection, thresholding, and quality-control criteria. In addition, large prospective multicenter studies linking presurgical fMRI findings with intraoperative mapping and standardized postoperative outcomes remain limited.

Advancing rs-fMRI from a promising adjunct to a broadly accepted clinical standard will require standardized acquisition and analysis procedures, further validation against reference standards including intraoperative mapping, and robust automated pipelines that deliver reproducible outputs in routine clinical workflows [139]. Until these steps are widely achieved, rs-fMRI should be adopted in practice as part of an integrated presurgical mapping toolbox rather than as the sole determinant of eloquence.

The main practical value of this review is therefore not to argue for replacement of established mapping techniques, but to provide a clinically oriented framework for selecting, combining, and interpreting tb-fMRI and rs-fMRI in common neurosurgical scenarios.

## Figures and Tables

**Table 1:** Common task-based fMRI (tb-fMRI) failure modes in neurosurgical candidates, the imaging/physiological mechanisms driving non-diagnostic or misleading maps, and practical mitigation strategies.

Failure Mode	Mechanism	Typical tb-fMRI Map Appearance	Practical Mitigation	Risk if Unrecognized
Inconsistent task performance	Patient lacks comprehension	Sparse or diffuse activation	Pre-scan task training, simplified paradigms, behavioral monitoring during scanning, repeated acquisitions if needed	Mislocalization leading to language deficits
Patient motion artifacts	Restlessness or inability to remain still	Blurred signals, false positives	Mock scanning when feasible, head stabilization, shorter or repeated runs, framewise displacement assessment, motion correction plus censoring/scrubbing criteria, reacquisition when QC is inadequate	Resection of functional areas
Incomplete motor task execution	Weak contraction or fatigue	Reduced or asymmetric activation	Passive motor paradigms, alternative motor tasks, behavioral or EMG monitoring when available, repeated runs, nTMS or intraoperative stimulation for confirmation	Postoperative motor deficits
Pediatric cooperation issues	Attention span limitations	Artifacts and poor signal	Age-appropriate tasks, mock scanner preparation, sleep/sedation-compatible rs-fMRI when task paradigms are not feasible, multimodal confirmation	Under-representation of functional areas
Tumor-related hemodynamic failure	Localized hemodynamic changes	Distorted activation patterns	CVR or breath-hold mapping, perfusion imaging, careful assessment of perilesional signal dropout, tractography, nTMS, and intraoperative stimulation as confirmatory methods	Resection of functional cortex

**Table 2:** At-a-glance synthesis of clinical evidence and practical implications discussed in Section 6.

Clinical Question	Evidence Is Strongest for	Main Practical Implication	Main Limitation
Cooperative adult with motor or language lesion near eloquent cortex	tb-fMRI, especially when task performance is reliable	Use tb-fMRI as first-line noninvasive mapping and integrate with tractography and neuronavigation	Task performance, thresholding, susceptibility artifacts, and NVU can reduce reliability
Sensorimotor mapping when task performance is limited	rs-fMRI sensorimotor network mapping	Add rs-fMRI, especially when voluntary movement is unreliable or impossible	Motion, preprocessing choices, and perilesional BOLD confounds still require QC
Language mapping	Multimodal interpretation of tb-fMRI, rs-fMRI, and intraoperative mapping	Use rs-fMRI as hypothesis-generating and supportive information, not as a standalone determinant of essential language sites	Language networks are distributed and more variable than sensorimotor networks
Suspected neurovascular uncoupling	CVR/perfusion-informed multimodal interpretation	Interpret absent or attenuated BOLD activation cautiously and consider CVR, perfusion imaging, tractography, nTMS, and intraoperative stimulation	Neither tb-fMRI nor rs-fMRI can fully exclude function in areas with abnormal hemodynamics
Non-cooperative, aphasic, pediatric, sedated, or cognitively impaired patients	rs-fMRI as task-free noninvasive mapping	rs-fMRI may serve as the primary noninvasive mapping approach when tb-fMRI is infeasible	Results should still be confirmed or contextualized with other modalities when clinically necessary

## Data Availability

No new data were created or analyzed in this study. Data sharing is not applicable to this article.

## Abbreviations

The following abbreviations are used in this manuscript:

BOLD	blood-oxygen-level-dependent
CT	computed tomography
CVR	cerebrovascular reactivity
DCS	direct cortical stimulation
DES	direct electrical stimulation
DICOM	Digital Imaging and Communications in Medicine
DMN	default mode network
DTI	diffusion tensor imaging
EOR	extent of resection
FIX	FMRIB’s ICA-based X-noiseifier
FLAIR	fluid-attenuated inversion recovery
fMRI	functional magnetic resonance imaging
GLM	general linear model
ICA	independent component analysis
MRI	magnetic resonance imaging
nTMS	navigated transcranial magnetic stimulation
NVU	neurovascular uncoupling
QC	quality control
RSN	resting-state network
SMN	sensorimotor network
rs-fMRI	resting-state functional magnetic resonance imaging
tb-fMRI	task-based functional magnetic resonance imaging
